# Effectiveness of electronic point-of-care reminders versus monthly feedback to improve adherence to 10 clinical recommendations in primary care: a cluster randomized clinical trial

**DOI:** 10.1186/s12911-019-0976-8

**Published:** 2019-11-29

**Authors:** Ermengol Coma, Manuel Medina, Leonardo Méndez, Eduardo Hermosilla, Manuel Iglesias, Carmen Olmos, Sebastian Calero

**Affiliations:** 1 Sistema d’Informació dels Serveis d’Atenció Primària (SISAP), Institut Català de la Salut. Institut Universitari d’Investigació en Atenció Primària Jordi Gol (IDIAP Jordi Gol), Barcelona, Spain; 2 Sistema de Informació pel Desenvolupament d’Investigació en Atenció Primària (SIDIAP), Institut Universitari d’Investigació en Atenció Primària Jordi Gol (IDIAP Jordi Gol), Barcelona, Spain; 3 Oficina Projecte ECAP, Centre de competència funcional, Institut Català de la Salut, Barcelona, Spain; 4 UGEAP Hospitalet Nord. DAP Delta. Institut Català de la Salut, Barcelona, Spain

**Keywords:** Reminder systems, Quality of health care, Primary health care, Electronic health record

## Abstract

**Background:**

Numerous studies have analyzed the effectiveness of electronic reminder interventions to improve different clinical conditions, and most have reported a small to moderate effect. Few studies, however, have analyzed reminder systems targeting multiple conditions, and fewer still have compared electronic point-of-care reminders systems with other forms of feedback designed to improve delivery of care.

**Methods:**

We performed an unblinded cluster randomized clinical trial to compare the effectiveness of an electronic point-of-care reminder system with that of a well-established system providing monthly feedback on adherence to clinical recommendations. The control group received monthly feedback only while the intervention group received monthly feedback in addition to on-screen point-of-care reminders for 10 clinical conditions. The study targeted all physicians and nurses at the 283 primary care centers managed by the Institut Català de la Salut (approximately 6600 professionals).

**Results:**

Following exclusions and randomization, 132 primary care centers (328,728 patients with reminders) were assigned to the intervention group while 137 centers (317,117 patients with reminders) were randomized to the control group. A 20.6% improvement (OR 1.29, 95% CI: 1.25–1.34) in reminder resolution rates was observed in the intervention group. Results varied according to the clinical condition. The most effective reminder was screening for diabetic retinopathy (OR 1.51, 95% CI:1.46–1.57) while the least effective reminders were measurement of glycated hemoglobin (OR: 1.10, 95% CI: 1.07–1.13) and smoking cessation encouragement (OR 1.12, 95% CI: 1.09–1.16).

**Conclusions:**

Electronic point-of-care reminders were more effective than the existing monthly feedback system at resolving the 10 clinical situations. However, more studies are needed to investigate the variations of the effect observed.

**Trial registration:**

Current Controlled Trials ISRCTN42391639, 08/10/2012. Retrospectively registered.

## Background

Reminders systems are used to prompt memory or help recall information. Examples of systems used in health care settings are computerized reminders, color code systems, telephone calls, and even letters and postcards [1]. The term *computer reminder* was used for the first time in 1976 by McDonald [2] in a clinical trial that found that computer-generated reminders improved clinician behavior. The effects of reminder systems have since been analyzed in many other trials and systematic reviews. Forty years after McDonald’s study, Cheung et al. [3] reviewed 35 systematic reviews on reminder systems published up to 2009 and assessed their quality using the AMSTAR tool. The results of their analyses, including those restricted to high-quality reviews (AMSTAR score > 5), showed that reminders were an effective means of improving clinician behavior and the quality of care delivery.

Reminders can target patients or clinicians. The most widely studied systems are probably those designed to improve prescribing practices and preventive care delivery. Electronic point-of-care reminders are reminders that are generated from patient data and delivered to the clinician during the clinical encounter. A Cochrane review by Shojania et al. [4] found that electronic reminders had a modest effect on process adherence (median improvement of 4.2%), while a systematic review and meta-analysis by Holt et al. [5] found an overall odds ratio of 1.79 (95% CI: 1.56–2.05) for computer-generated on-screen and paper reminders. Most studies of the effectiveness of reminders in primary care settings have analyzed systems targeting a single disorder or clinical condition [6–13]. The few studies of multiple reminder interventions have mostly focused on vaccination, screening, and adverse drug events [14, 15].

In a systematic review aimed at identifying features of successful reminder systems, Kawamoto et al. [16] found that effective systems 1) provided automatic decision support, 2) used computers to do this, 3) provided a recommendation rather than just an assessment, and 4) provided the support at the time and place of the decision-making.

The *Institut Català de la Salut* (ICS) is the main primary care service provider in Catalonia and manages approximately 80% of all primary care teams in the Catalan public health system. In 2005, the ICS implemented a universal electronic medical record system for use in primary care known as the ECAP (*Estació Clínica d’Atenció Primària*). The ECAP is a software system that serves as a repository for structured data on diagnoses (coded according to the International Classification of Diseases 10th revision), clinical variables, prescription data, and laboratory test results. Every month, thanks to a system that has been in place since 2006, primary health care professionals (HCPs) receive a report on their performance through the ECAP. Performance is calculated according to relevant clinical indicators and the report contains feedback and a list of patients with a clinical situation that can be improved [17, 18]. The clinical indicators, which are based on scientific evidence, form what is known as the EQA (*Estàndard de Qualitat Assistencial*) [17], which is a quality measure similar to the Quality Outcomes Framework developed by the UK National Health Services. HCPs can access these monthly reports and lists whenever they wish through the ECAP menu. The EQA is also used in the ICS’s pay for performance program.

EQA results have gradually improved over the years, and there is evidence linking improvements to an increasing use of the system by HCPs [17]. To further develop the feedback system in place, we designed an electronic point-of-care reminder system incorporating the success factors identified by Kawamoto et al. [16]. This system is integrated into the ECAP and displays the reminders during the clinical encounter. The hypothesis was that a synchronous decision support system available to HCPs at the point of care would improve adherence to clinical recommendations. To test this hypothesis before deployment of the system, we designed a clinical trial to determine whether immediate, point-of-care, electronic reminders were more effective than the monthly feedback system already in place.

## Methods

### Study design

We designed an unblinded cluster randomized trial to compare the effectiveness of an electronic point-of-care reminder system with that of a well-established feedback system to improve adherence to clinical recommendations. The study was originally designed to last one year with a revision after 6 month. The study was, finally, conducted between February 1 and July 31, 2012 and was registered at http://www.controlled-trials.com (ISRCTN42391639). The study protocol was published in 2016 [19]. We adhere to CONSORT guidelines for reporting of results.

### Study population

The eligible study population comprised all primary care family physicians and nurses (referred to hereafter as HCPs) delivering care to people aged over 14 years at the 283 primary care centers managed by the ICS. HCPs from eight primary care centers participating in another clinical trial on electronic reminders were excluded, as were those from the two centers that had participated in the pilot phase of the trial.

### Intervention

We designed reminders for 10 improvable clinical situations corresponding to a chronic condition or a preventive care measure within the EQA indicator [17]. Computer algorithms were used to analyze the ECAP data weekly and identify patients aged over 14 years with at least one of the 10 clinical situations. The reminders were automatically displayed in a pop-up window that appeared when the HCP accessed the patient’s medical record. Each pop-up window listed the clinical alert(s) generated for the patient, made a recommendation in the form of a call for action (e.g., measure blood pressure) and, where appropriate, showed clinical values and corresponding dates. Each pop-up reminder was accompanied by an orange or red icon indicating the importance of the recommendation (Table 1).

**Table 1:** Clinical recommendations, conditions and type of reminder

Recommendation	Clinical condition	Type of reminder
Moderately important (orange icon)		
Measure blood pressure: ≤140/90 or ≤ 150/95 depending on risk status	High blood pressure/high cardiovascular risk	Measurement
Measure glycated hemoglobin: HbA1c ≤8%	Type 2 diabetes mellitus	Measurement
Measure cholesterol: LDL <120	Cerebrovascular accident/ischemic heart disease	Measurement
Screen for diabetic retinopathy	Type 2 diabetes mellitus	Screening
Encourage smoking cessation	Smoking	Lifestyle
Provide Hepatitis B vaccination to patients with Hepatitis C	Hepatitis C	Vaccination
Very important (red icon)		
Treat with ACEI/ARB	Heart failure	Treatment
Treat with beta blockers	Heart failure/ischemic heart disease	Treatment
Treat with antiplatelet drug/anticoagulant	Atrial fibrillation	Treatment
Treat with antiplatelet drug	Cerebrovascular accident/ischemic heart disease	Treatment

Abbreviations: *ACEI* Angiotensin-converting enzyme inhibitor, *ARB* Angiotensin II receptor blocker, *LDL* Low-density lipoprotein

### Control group

**Fig. 1:**
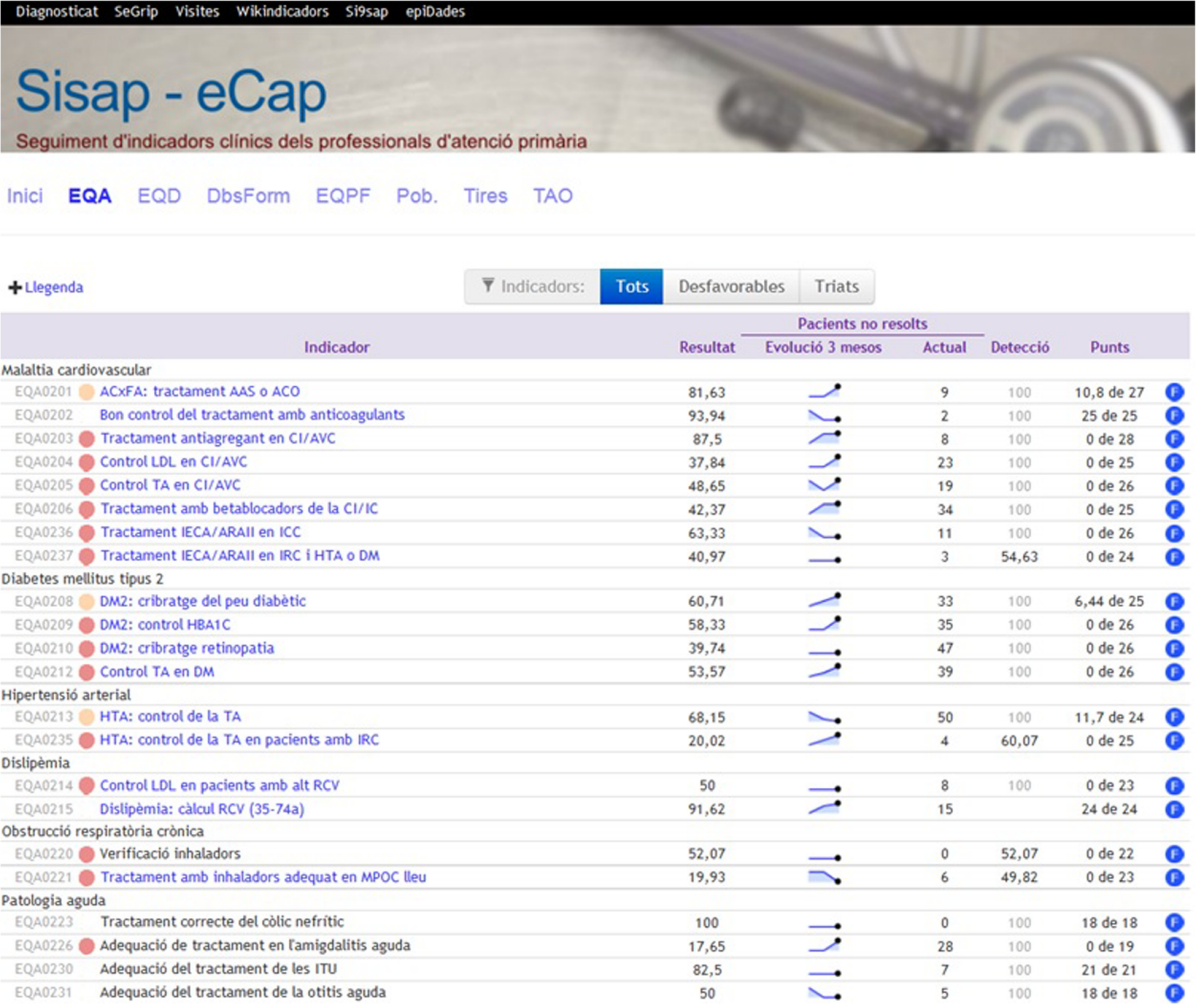
Screenshot of feedback screen that can be accessed by primary health care professionals within the centralized electronic medical records system. Well-established feedback without reminders (control group)

All the participants in the trial, including those in the control group, continued to receive feedback through the usual system. This consisted of a monthly update for each HCP specifying their rate of adherence to the different EQA recommendations together with comparative figures for previous months and their environment (Fig. 1). The report was accompanied by a list of noncompliant patients. An online and previous version of the feedback system can be consulted at http://www.amf-semfyc.com/sisap/ [18].

The main differences between the well-stablished feedback and the electronic reminders are shown in Table 2.

**Table 2:** Differences between well-established feedback and electronic reminders

	Established feedback	Reminders
Periodicity	Monthly	Weekly
Trigger	Professional initiative	Clinical encounter
What	61 recommendations	10 recommendations
When	Off-line	Online
Where	Web report	Reminder at the point-of-care
Configurability	No	Yes (in one subgroup)
Integration with electronic health record functionality	Low	High

### Intervention groups

There were three types of electronic reminders and hence three intervention groups. The reminders were incremental and consisted of 1) a pop-up window, 2) a pop-up window plus a schedule icon, and 3) a pop-up window plus a schedule icon plus a reminder filter settings option.

### Pop-up window

In the pop-up window group, HCPs accessing the medical record of a patient with an improvable clinical situation were presented with a pop-up window. To consult more information in the patient’s record, the HCP first had to close the window or click on the reminder link.

### Pop-up window + schedule icon

In addition to the pop-up window, HCPs in the pop-up window + schedule icon group saw an orange or red icon next to the names of the patients with reminders in the day’s schedule. They were thus immediately able to see how many patients had a reminder on a given day. The icons were orange or red depending on whether the situation was moderately or very important and they also contained a number showing how many reminders each patient had.

### Pop-up window + schedule icon + filter settings option

In this third group, in addition to the pop-up window and schedule icon, HCPs were able to apply filters to choose which type of reminders and which patients they wanted to see information for. The default setting for all three groups was “all reminders”.

### Randomization

**Fig. 2:**
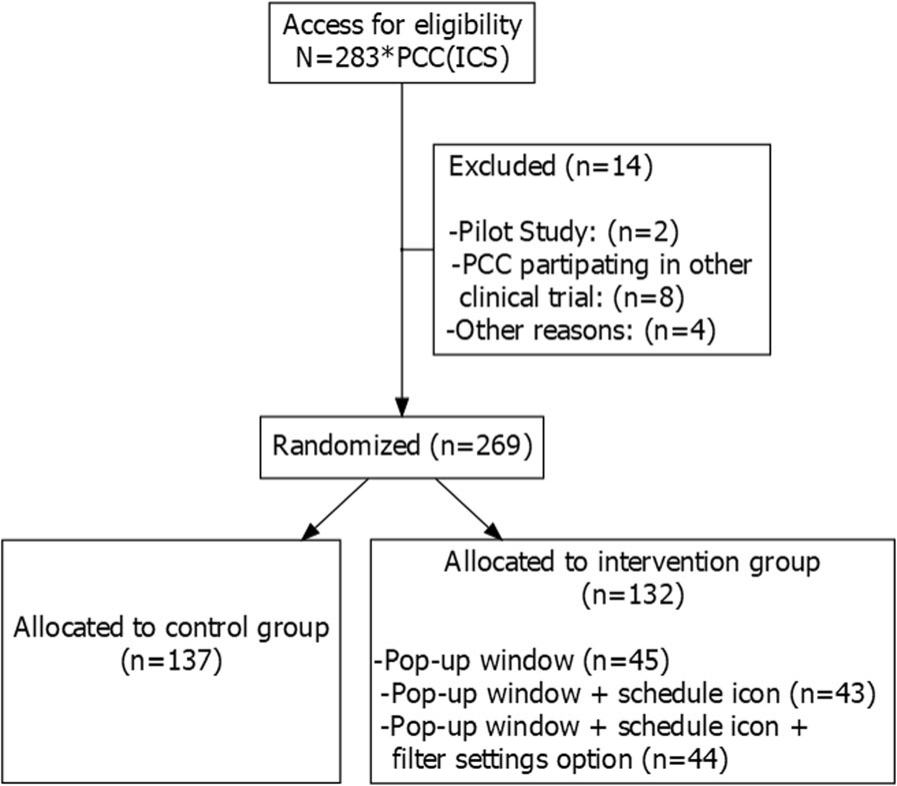
Workflow of study population

The units of analysis were the primary care centers and therefore all HCPs from the same center were randomized to the same arm. Randomization was performed using the Stata/SE module to design randomized controlled trials (v. 11.2, StataCorp) [20]. In order to achieve a balanced distribution of the quality of care at the different centers in each arm, these were stratified into quintiles according to EQA results from the month prior to the intervention. The workflow of study population is shown in Fig. 2.

### Sample size

A 10% difference in resolution of clinically improvable situations between groups was considered clinically relevant. Assuming a resolution rate of 59% in the intervention group [21], an alpha error of 0.01, a power of 95%, and a loss to follow-up of 10%, and correcting for an intraclass correlation of 0.05 in the clusters (primary care centers), it was calculated that 15,950 reminders would be required for each of the four groups (total, 63,800). Nevertheless, in view of the design of the computerized tool, the cluster randomization design, and the distribution of the sample, it was decided not to limit the study to the calculated sample size.

### Outcome and variables

The primary outcome measure was the resolution of reminders. A reminder was considered to be resolved when the clinically improvable condition for which it was generated was resolved. Solutions varied according to the type of reminder, and included, for example, prescription of a drug, implementation of a preventive measure, or achievement of a clinical or laboratory target.

The other study variables were the characteristics of the HCPs and the primary care centers to which they belonged. The characteristics analyzed for each center were certification to train postgraduate students, rural or urban location, population assigned and attended, mean number of visits per patient per year, and mean number of physicians and nurses.

### Statistical analysis

Because of the cluster randomization design, we used statistical techniques that took into account the correlations between patients in the different clusters. Intervention effect was estimated using hierarchical (multilevel) models (Wald test), with reminder at level 1 and HCP at level 2. Logistic regression models were used to estimate the effect of the intervention first with adjustment for random HCPs only and then with the addition of EQA results. We finally estimated whether the effect was modified by the introduction of variables at the reminder level. A subgroup analysis by type of reminder was also performed.

Statistical significance was set at 5%. The analyses were performed in Stata/SE, version 11.2 (StataCorp).

## Results

### Baseline results

The baseline analysis identified 645,845 patients with an improvable clinical situation (13% of the population > 14 years) and generated 968,272 reminders, which is the equivalent of 1.49 reminders per patient.

In the randomization process, 132 primary care centers delivering care to 328,728 patients with 488,811 reminders were assigned to the intervention group, while 137 HCPs delivering care to 317,117 patients with 469,514 reminders were assigned to the control group.

The intervention and control groups were comparable in terms of baseline patient and primary care center characteristics (Table 3).

**Table 3:** Baseline characteristics of primary care centers and patients with reminders in the control and intervention groups

	Control group	Intervention group	*P* value
Patients with reminders	317,117	328,728	
Mean age	66.38	66.48	0.0047
95% CI	(66.33–66.43)	(66.43–66.53)	
% women	46.15	46.29	0.235
Mean no. of reminders	1.48	1.49	0.0012
95% CI	(1.47–1.48)	(1.48–1.49)	
Mean no. of visits per patient	1.69	1.75	0.0000
95% CI	(1.68–1.70)	(1.74–1.75)	
Number of primary care centers	137	132	
% rural centers	36.5	46.2	0.11
% of teaching centers	21.9	25.76	0.477
Mean no. of patients >14 assigned	16,279.81	17,429.42	0.2377
95% CI	(14,920.39–17,639.23)	(16,072.79–18,786.04)	
% population served	70.7	71.23	0.4716
95% CI	(69.69–71.74)	(70.26–72.19)	
Mean no. of patients per general practitioner	1337.21	1373.31	0.2565
95% CI	(1296.99–1377.43)	(1324.85–1421.77)	
Frequency of visits per patient per year	6.85	6.99	0.4669
95% CI	(6.59–7.11)	(6.72–7.27)	
Mean no. of general practitioners per center	11.97	12.52	0.3943
95% CI	(11.05–12.89)	(11.65–13.39)	
Mean no. of nurses per center	12.73	13.18	0.4899
95% CI	(11.78–13.67)	(12.27–14.1)	

**Fig. 3:**
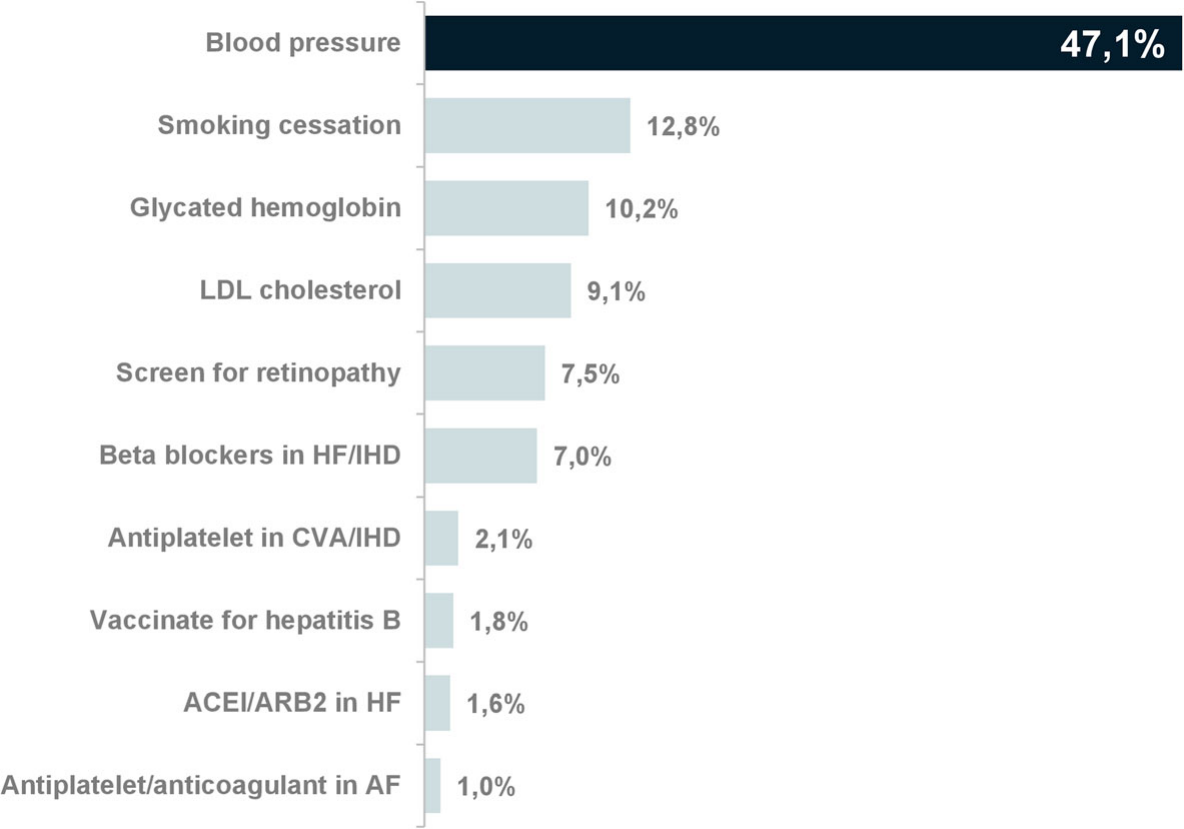
Percentage of reminders by clinical recommendation. Subtitle: Almost half of the reminders were to measure blood pressure. Abbreviations for Fig. 3: LDL: low-density lipids; HF: heart failure; IHD: ischemic heart disease; CVA: Cerebrovascular accident; ACEI: angiotensin-converting enzyme inhibitor; ARB: angiotensin II receptor blocker; AF: atrial fibrillation

Almost half of the reminders (47%) were to measure blood pressure (Fig. 3). The next two most common reminders were to encourage smoking cessation (12.8%) and measure glycated hemoglobin in patients with type 2 diabetes mellitus (10.2%). Table 4 shows the number and percentage of reminders by type and clinical condition in the intervention and control groups.

**Table 4:** Distribution of reminders by clinical condition and specific reminder in the intervention and control groups

		Control group reminders		Intervention group reminders		Total reminders	
Clinical condition	Type of reminder	No.	%	No.	%	No.	%
Type 2 diabetes mellitus	Screen for diabetic retinopathy	34,748	7.4	36,649	7.5	71,397	7.45
	Measure glycated hemoglobin	47,582	10.13	49,873	10.2	97,455	10.17
High blood pressure	Measure blood pressure	22,0382	46.94	23,0559	47.17	45,0941	47.05
Atrial fibrillation	Treat with antiplatelet drug/anticoagulant	4650	0.99	4852	0.99	9502	0.99
Cerebrovascular accident/ischemic heart disease	Measure LDL cholesterol	43,032	9.17	44,196	9.04	87,228	9.1
	Treat with antiplatelet drug	9807	2.09	10,489	2.15	20,296	2.12
Heart failure/ischemic heart disease	Treat with beta blocker	32,810	6.99	34,312	7.02	67,122	7
Heart failure	Treat with ACEI/ARB	7189	1.53	7733	1.58	14,922	1.56
Hepatitis C	Hepatitis B vaccine	8681	1.85	8535	1.75	17,216	1.8
Smoking	Encourage smoking cessation	60,633	12.91	61,613	12.6	122,246	12.76
	Total	469,514	100	488,811	100	958,325	100

Abbreviations: *ACEI* Angiotensin-converting enzyme inhibitor, *ARB* Angiotensin II receptor blocker, *LDL* Low-density lipoprotein

### Outcome and estimation

The reminder resolution rate was 27.5% for the intervention group and 22.8% for the control group. Overall, the intervention led to a 20.6% improvement (OR 1.29, 95% CI: 1.25–1.34) in adherence to clinical recommendations. The differences between the intervention groups were nonsignificant, but all three rates were higher than the rate in the control group and they also showed a tendency to increase with the addition of reminder strategies (Table 5).

**Table 5:** Resolution of reminders by type of intervention

	Reminders at baseline	Not resolved	Resolved	% Resolved	Increase (%)	OR (95% CI)	*P*-value
Control group	469,514	362,542	106,972	22.8		1 (Reference)	
Intervention	488,811	354,397	134,414	27.5	20.6	1.29 (1.25–1.34)	<0.001
Pop-up window	164,006	119,972	44,034	26.8	17.5	1.24 (1.17–1.30)	<0.001
Above + schedule icon	154,945	111,802	43,143	27.8	21.9	1.31 (1.24–1.38)	<0.001
Above + filter settings options	169,860	122,361	47,499	28.0	22.8	1.33 (1.27–1.40)	<0.001
Total	958,325	716,939	241,386	25.2			

Statistically significant differences were observed in all reminders. However, results varied according to the clinical condition The two reminders associated with the highest resolution rates (>30%) were the reminders to administer anticoagulant/antiplatelet treatment and measure blood pressure. The lowest rates were seen for hepatitis B vaccination and smoking cessation encouragement (Table 6).

**Table 6:** Resolution rates in control and intervention groups by clinical condition and specific reminders

		Resolution (%)			OR (95% CI)
Clinical condition	Type of reminder	Total	Control group	Intervention group	
Type 2 diabetes mellitus	Screen for diabetic retinopathy	23.88	20.05	27.52	1.51 (1.46–1.57)
	Measure glycated hemoglobin	26.23	25.32	27.11	1.1 (1.07–1.13)
Heart failure/ischemic heart disease	Treat with beta blocker	14.64	12.58	16.61	1.38 (1.32–1.44)
Heart failure	Treat with ACEI/ARB	24.53	21.51	27.35	1.37 (1.27–1.48)
High blood pressure	Measure blood pressure	30.19	27	33.24	1.35 (1.33–1.36)
Hepatitis C	Hepatitis B vaccine	5.59	4.99	6.2	1.26 (1.1–1.43)
Atrial fibrillation	Treat with antiplatelet drug/anticoagulant	31.41	28.97	33.76	1.25 (1.15–1.36)
Cerebrovascular accident/ischemic heart disease	Treat with antiplatelet drug	33.32	30.75	35.72	1.25 (1.18–1.32)
	Measure LDL cholesterol	27.19	25.71	28.63	1.16 (1.13–1.19)
Smoking	Encourage smoking cessation	12.04	11.42	12.66	1.12 (1.09–1.16)

Abbreviations: *ACEI* Angiotensin-converting enzyme inhibitor, *ARB* Angiotensin II receptor blocker, *LDL* Low-density lipoprotein

The most effective reminder (when comparing control and intervention groups) was the diabetic retinopathy screening reminder (OR: 1.51, 95% CI: 1.46–1.57) and the two least effective ones were the reminders to measure glycated hemoglobin (OR 1.1, 95% CI: 1.07–1.13) and encourage smoking cessation (OR: 1.12, 95% CI: 1.09–1.16).

On analyzing the reminders by type (treatment, measurement, screening, vaccination, and lifestyle), measurement reminders had the highest resolution frequencies but the most effective ones were for screening and treatment (Table 7).

**Table 7:** Resolution rates by type of reminder in the control and intervention groups

	Resolution (%)		OR (95% CI)
Type of reminder	Control group	Intervention group	
Screening	20.05	27.52	1.51 (1.46–1.57)
Measurement	26.56	31.67	1.28 (1.27–1.29)
Treatment	18.43	23	1.32 (1.28–1.36)
Vaccination	4.99	6.2	1.25 (1.1–1.43)
Lifestyle	11.42	12.66	1.12 (1.09–1.16)

The multivariate analyses adjusted for age and sex, type of reminder, and EQA results showed no significant differences for the estimated effect of the intervention.

## Discussion

The electronic on-screen point-of-care reminder intervention analyzed in our study had an effectiveness rate of 20.6%, which is higher than most rates reported to date. A systematic review of 61 clinical trials evaluating reminder systems for preventive care measures reported an average improvement of 12 to 14% [22]. However, in a slightly later Cochrane review of the effect on-screen reminders [4] Shojaina et al. found an improvement of 4.2% (IQR, 0.8–18.8%), suggesting considerable variability across studies. According to the overview of systematic reviews by Cheung et al. [3] and the updated Cochrane review [23], reminder systems continue to have a small or moderate effect.

Our study has several distinguishing features that could account in part for the higher effectiveness rate observed.

We analyzed reminders for different types of clinical conditions. Few of the studies published to date have analyzed the use of simultaneous reminders for more than one condition or situation [10, 15, 24, 25]. One of these studies (perhaps the one that is most similar to ours) analyzed the impact of computerized reminders for 13 clinical situations and reported an effectiveness rate of 10% [21]. The relatively high rate observed in our study is even more noteworthy considering that one of the recommendations for increasing the effectiveness of reminder systems is to limit the number of alerts used [26], although optimal numbers have yet to be investigated. In our case at least, the generation of reminders for 10 improvable clinical situations (with a mean of 1.49 reminders per patient) was not found to be a barrier to uptake.

Our findings are based on an analysis of almost 1 million weekly reminders generated for over 600,000 patients. Most of the studies to date have analyzed much smaller populations [21].

The vast majority of studies to date have compared reminder or feedback interventions with no interventions. In our study, the HCPs in the control group continued to receive monthly feedback within a widely used system that had been in place since 2006 [18]. One of the research needs identified in the 2012 Cochrane review was the design of studies comparing reminder systems with other types of feedback systems [23].

The HCPs in our study were very used to receiving and using feedback, with data from 2007 showing that approximately 71% of physicians consulted their feedback reports every month [17]. In other studies, absence of a significant effect has been attributed to the fact that the reminder systems tested were used by only a minority of the target population [27].

One might expect that some of the distinctive features of our study design, such as a control group used to receiving feedback, would have reduced the likelihood of finding differences between the groups, but this was not the case. One possible explanation for the larger effect observed in our study is that when designing the system we took into account the criteria for success identified by Kawamoto et al. [16] and others. The 2012 Cochrane review by Ivers et al. [23] reported that effectiveness also depended on baseline performance and on how feedback is given. In particular, the authors concluded that feedback was more effective when given by a supervisor or a colleague, when given both verbally and in writing, when given more than once, and when given together with clear targets and an action plan for achieving these. Other authors have found reminders to be more effective when feedback is given regularly, when it includes information on the right solution, and when the reminders are integrated into the clinician’s workflow [12, 26, 28].

In our study, recommendations were delivered to the HCPs weekly rather than monthly as in the existing feedback system. In addition, reminders were generated automatically, shown during the clinical encounter, accompanied by additional information and recommendations, and based on indicators that had been in use for years, increasing thus the likelihood of widespread uptake.

We found no significant differences in effect between the three intervention groups. Although one might have expected the reminder system featuring the filter options to have been associated with better performance due to its greater flexibility, the main differences observed were between the simple pop-up window group and the control group. More studies are probably needed to analyze the use of customizable reminder settings.

Generally speaking, the reminders were effective for all the clinical conditions analyzed, although there were some differences. We observed a larger effect for clear, easy-to-resolve reminders, such as prompts to screen for diabetic retinopathy, and for reminders about potentially serious situations, such as antiplatelet or anticoagulant treatment in patients with heart disease. The reminders with the smallest effect were those related to more complicated or difficult-to-resolve situations, such as smoking cessation, which is a difficult goal to achieve in the short term. Our findings in this regard are consistent with reports that feedback on familiar, acceptable tasks or designed to improve prescribing practices are more effective that other types of feedback [23]. One possible explanation could be that when faced with multiple reminders, clinicians prioritize those that are clinically more important or that can be resolved with less time or effort [23, 26, 29]. This hypothesis should be tested in studies analyzing the effect of simultaneous reminders and the reasons behind prioritization.

Our study has certain limitations. While some authors have suggested that pay-for-performance systems might lead more to improvements in documentation than in care delivery [30, 31], a Cochrane review on the topic concluded that there was insufficient evidence for or against financial incentives having a positive impact on the quality of care in primary settings [32]. In our study, as described in the protocol [19], the financial rewards associated with the EQA indicator were low and were applicable to the HCPs in both the intervention and control groups. We therefore do not believe that they will have influenced the results.

It was not possible to blind the HCPs to the group assignment and this may have resulted in some bias. Nonetheless, we selected cluster randomization because most studies of this type use this design; we decided against any design in which the same HCP would have some patient records with reminders and others without. In a study by Chambers et al. [33] on the effect of computerized reminders on vaccination rates in primary care, the physicians were randomized to three arms: one that received no reminders, another that received reminders for all their patients, and another that received reminders for half their patients. The worst performance was observed in the group that received sporadic reminders, suggesting that physicians might come to rely on reminders and in their absence assume that a patient does not need a particular intervention.

The duration of our intervention, 6 months, may have limited our findings in two ways. First, the effect of reminders related to situations that require a longer resolution time might have been diminished. One example is the reminder to encourage patients to quit smoking, which was one of the least effective reminders in our study. Second, the study period may have been too short to detect “reminder fatigue”, limiting thus our ability to estimate long-term effects. Failure to analyze long-term effects is one of the main limitations identified in reviews of reminder system studies [3]. Why then did we stop our study after 6 months when the protocol specified 12 months with an interim analysis at 6 months [19]? On observing the significant differences between the intervention and control groups at the interim point, considering the health implications, and since no other harm were detected, we decided to discontinue the study and implement the intervention across all the primary care centers. More studies are needed to determine whether the effect observed for the intervention is maintained in time, although such studies are difficult to conduct in everyday practice. One of the main advantages of electronic reminder systems and one that is of particular interest in primary care is that even though these systems do not always have a large effect, they are relatively cheap and easy to manage [3]. This advantage, however, is also a potential stumbling block because as the system is relatively easy to apply (technically speaking, it costs the same to produce results for 1 HCP as for 6000), there is a risk that systems with even the least hint of success could be deployed without the necessary guarantees.

## Conclusions

The results of this cluster randomized trial show that an electronic point-of-care reminder system featuring simultaneous reminders on multiple clinical conditions can have a positive effect. They also show that immediate reminders are more effective than periodic feedback, an important consideration for future decision support interventions. We observed variations in the effect of the different reminders used and these should be investigated in further studies, alongside other factors such as long-term effects or the effects of multiple reminders and customizable systems.

## Data Availability

The datasets used and/or analyzed during the current study are available from the corresponding author on reasonable request.

## Abbreviations

ACEI: angiotensin-converting enzyme inhibitors
AF: atrial fibrillation
ARB: angiotensin II receptor blocker
CVA: cerebrovascular accident
ECAP: *Estació Clínica d’Atenció Primària* (primary care electronic medical record system managed by the *Institut Català de la Salut*)
ECRC: Ethics and Clinical Research Committee
EQA: *Estàndard de Qualitat Assistencial* (quality of care indicator)
HbA1c: glycated hemoglobin
HCP: health care professional
HF: heart failure
ICS: *Institut Català de la Salut (*Catalan Institute of Health*)*
IHD: ischemic heart disease
LDL: low-density lipids
SIDIAP: *Sistema de Informació pel Desenvolupament d’Investigació en Atenció Primària (*information system for research in primary care)
SISAP: *Sistema d’Informació dels Serveis d’Atenció Primària* (primary care information system managed by the *Institut Català de la Salut* ICS)

## References

[1] MeSH Browser (2011 MeSH). Bethesda (MD): National Library of Medicine (US), Reminder Systems; [Cited 2017 Aug]. [Internet]. Available from: https://www.ncbi.nlm.nih.gov/mesh?Db=mesh&term=Reminder+Systems

[2] Mc Donald CJ. Use of a computer to detect and respond to clinical events: its effect on clinician behavior. Ann Intern Med. 1976;84(2):162–7. [Cited 1976 Feb 1] Available from: http://www.ncbi.nlm.nih.gov/pubmed/1252043 10.7326/0003-4819-84-2-162 1252043

[3] Cheung A, Weir M, Mayhew A, Kozloff N, Brown K, Grimshaw J. Overview of systematic reviews of the effectiveness of reminders in improving healthcare professional behavior. Systematic reviews [Internet]. 2012 Aug 16 [cited 2012 Aug 16];1:36. Available from: http://www.ncbi.nlm.nih.gov/pubmed/22898173 10.1186/2046-4053-1-36 PMC3503870 22898173

[4] Shojania KG, Jennings A, Mayhew A, Ramsay CR, Eccles MP, Grimshaw J. The effects of on-screen, point of care computer reminders on processes and outcomes of care. The Cochrane database of systematic reviews [Internet]. 2009 Jul 8 [cited 2009 Jul 8];(3):CD001096. Available from: http://www.ncbi.nlm.nih.gov/pubmed/19588323 10.1002/14651858.CD001096.pub2 PMC4171964 19588323

[5] Holt TA, Thorogood M, Griffiths F. Changing clinical practice through patient specific reminders available at the time of the clinical encounter: systematic review and meta-analysis. Journal of general internal medicine [Internet]. 2012 Aug 10 [cited 2012 Aug 10];27(8):974–84. Available from: http://www.ncbi.nlm.nih.gov/pubmed/22407585 10.1007/s11606-012-2025-5 PMC3403145 22407585

[6] Eccles M, McColl E, Steen N, Rousseau N, Grimshaw J, Parkin D, et al. Effect of computerised evidence based guidelines on management of asthma and angina in adults in primary care: cluster randomised controlled trial. BMJ (Clinical research ed) [Internet]. 2002 Oct 26 [cited 2002 Oct 26];325(7370):941. Available from: http://www.ncbi.nlm.nih.gov/pubmed/12399345 10.1136/bmj.325.7370.941 PMC130060 12399345

[7] Filippi A, Sabatini A, Badioli L, Samani F, Mazzaglia G, Catapano A, et al. Effects of an automated electronic reminder in changing the antiplatelet drug-prescribing behavior among Italian general practitioners in diabetic patients: an intervention trial. Diabetes care [Internet]. 2003 May 1 [cited 2003 May 1];26(5):1497–500. Available from: http://www.ncbi.nlm.nih.gov/pubmed/12716811 10.2337/diacare.26.5.1497 12716811

[8] Hicks LS, Sequist TD, Ayanian JZ, Shaykevich S, Fairchild DG, Orav EJ, et al. Impact of computerized decision support on blood pressure management and control: a randomized controlled trial. Journal of general internal medicine [Internet]. 2008 Apr 1 [cited 2008 Apr 1];23(4):429–41. Available from: http://www.ncbi.nlm.nih.gov/pubmed/18373141 10.1007/s11606-007-0403-1 PMC2359515 18373141

[9] Krall MA, Traunweiser K, Towery W. Effectiveness of an electronic medical record clinical quality alert prepared by off-line data analysis. Studies in health technology and informatics [Internet]. 2004 [cited 2004];107(Pt 1):135–9. Available from: http://www.ncbi.nlm.nih.gov/pubmed/15360790 15360790

[10] Sequist TD, Gandhi TK, Karson AS, Fiskio JM, Bugbee D, Sperling M, et al. A randomized trial of electronic clinical reminders to improve quality of care for diabetes and coronary artery disease. Journal of the American Medical Informatics Association: JAMIA [Internet]. 2005 Aug 31 [cited 2005 Aug 31];12(4):431–7. Available from: http://www.ncbi.nlm.nih.gov/pubmed/15802479 10.1197/jamia.M1788 PMC1174888 15802479

[11] Tierney WM, Overhage JM, Murray MD, Harris LE, Zhou X-H, Eckert GJ, et al. Effects of computerized guidelines for managing heart disease in primary care. Journal of general internal medicine [Internet]. 2003 Dec 1 [cited 2003 Dec 1];18(12):967–76. Available from: http://www.ncbi.nlm.nih.gov/pubmed/14687254 10.1111/j.1525-1497.2003.30635.x PMC1494965 14687254

[12] van Wyk JT, van Wijk MAM, Sturkenboom MCJM, Mosseveld M, Moorman PW, van der Lei J. Electronic alerts versus on-demand decision support to improve dyslipidemia treatment: a cluster randomized controlled trial. Circulation [Internet]. 2008 Jan 22 [cited 2008 Jan 22];117(3):371–8. Available from: http://www.ncbi.nlm.nih.gov/pubmed/18172036 10.1161/CIRCULATIONAHA.107.697201 18172036

[13] Guiriguet C, Muñoz-Ortiz L, Burón A, Rivero I, Grau J, Vela-Vallespín C, et al. Alerts in electronic medical records to promote a colorectal cancer screening programme: a cluster randomised controlled trial in primary care. The British journal of general practice: the journal of the Royal College of General Practitioners [Internet]. 2016 Jul 6 [cited 2016 Jul 6];66(648):e483–90. Available from: http://www.ncbi.nlm.nih.gov/pubmed/27266861 10.3399/bjgp16X685657 PMC4917051 27266861

[14] Tamblyn R, Huang A, Perreault R, Jacques A, Roy D, Hanley J, et al. The medical office of the 21st century (MOXXI): effectiveness of computerized decision-making support in reducing inappropriate prescribing in primary care. CMAJ: Canadian Medical Association journal = journal de l’Association medicale canadienne [Internet]. 2003 Sep 16 [cited 2003 Sep 16];169(6):549–56. Available from: http://www.ncbi.nlm.nih.gov/pubmed/12975221 PMC191278 12975221

[15] Frank O, Litt J, Beilby J. Opportunistic electronic reminders. Improving performance of preventive care in general practice. Australian family physician [Internet]. 2004 Feb 1 [cited 2004 Feb 1];33(1–2):87–90. Available from: http://www.ncbi.nlm.nih.gov/pubmed/14988972 14988972

[16] Kawamoto K, Houlihan CA, Balas EA, Lobach DF. Improving clinical practice using clinical decision support systems: a systematic review of trials to identify features critical to success. BMJ (Clinical research ed) [Internet]. 2005 Apr 2 [cited 2005 Apr 2];330(7494):765. Available from: http://www.ncbi.nlm.nih.gov/pubmed/15767266 10.1136/bmj.38398.500764.8F PMC555881 15767266

[17] Coma E, Ferran M, Méndez L, Iglesias B, Fina F, Medina M. Creation of a synthetic indicator of quality of care as a clinical management standard in primary care. SpringerPlus [Internet]. 2013 Dec 13 [cited 2013 Dec 13];2(1):51. Available from: http://www.ncbi.nlm.nih.gov/pubmed/23450738 10.1186/2193-1801-2-51 PMC3581769 23450738

[18] Coma E, Méndez L. SISAP: 4 años buceando en mares de datos (AMF 2010) Experiencias para compartir. AMF [Internet]. 2010 [cited 2010];(8):473–6. Available from: http://amf-semfyc.com/web/article_ver.php?id=132

[19] Méndez Boo L, Coma E, Medina M, Hermosilla E, Iglesias M, Olmos C, et al. Effectiveness of computerized point-of-care reminders on adherence with multiple clinical recommendations by primary health care providers: protocol for a cluster-randomized controlled trial. SpringerPlus [Internet]. 2016 Sep 7 [cited 2016 Sep 7];5(1):1505. Available from: http://www.ncbi.nlm.nih.gov/pubmed/27652078 10.1186/s40064-016-3124-2 PMC5014773 27652078

[20] 1990. Ryan P. RALLOC: Stata module to design randomized controlled trials. [Internet]. Available from: https://ideas.repec.org/c/boc/bocode/s319901.html

[21] Demakis JG, Beauchamp C, Cull WL, Denwood R, Eisen SA, Lofgren R, et al. Improving residents’ compliance with standards of ambulatory care: results from the VA Cooperative Study on Computerized Reminders. JAMA [Internet]. 2000 Sep 20 [cited 2000 Sep 20];284(11):1411–6. Available from: http://www.ncbi.nlm.nih.gov/pubmed/10989404 10.1001/jama.284.11.1411 10989404

[22] Dexheimer JW, Talbot TR, Sanders DL, Rosenbloom ST, Aronsky D. Prompting clinicians about preventive care measures: a systematic review of randomized controlled trials. Journal of the American Medical Informatics Association: JAMIA [Internet]. 2008 Jun 28 [cited 2008 Jun 28];15(3):311–20. Available from: http://www.ncbi.nlm.nih.gov/pubmed/18308989 10.1197/jamia.M2555 PMC2410011 18308989

[23] Ivers N, Jamtvedt G, Flottorp S, Young JM, Odgaard-Jensen J, French SD, et al. Audit and feedback: effects on professional practice and healthcare outcomes. The Cochrane database of systematic reviews [Internet]. 2012 Jun 13 [cited 2012 Jun 13];(6):CD000259. Available from: http://www.ncbi.nlm.nih.gov/pubmed/22696318 10.1002/14651858.CD000259.pub3 PMC11338587 22696318

[24] Overhage JM, Tierney WM, McDonald CJ. Computer reminders to implement preventive care guidelines for hospitalized patients. Archives of internal medicine [Internet]. 1996 Jul 22 [cited 1996 Jul 22];156(14):1551–6. Available from: http://www.ncbi.nlm.nih.gov/pubmed/8687263 8687263

[25] Dexter PR, Perkins S, Overhage JM, Maharry K, Kohler RB, McDonald CJ. A computerized reminder system to increase the use of preventive care for hospitalized patients. The New England journal of medicine [Internet]. 2001 Sep 27 [cited 2001 Sep 27];345(13):965–70. Available from: http://www.ncbi.nlm.nih.gov/pubmed/11575289 10.1056/NEJMsa010181 11575289

[26] Saleem JJ, Patterson ES, Militello L, Render ML, Orshansky G, Asch SM. Exploring barriers and facilitators to the use of computerized clinical reminders. Journal of the American Medical Informatics Association: JAMIA [Internet]. 2005 Aug 31 [cited 2005 Aug 31];12(4):438–47. Available from: http://www.ncbi.nlm.nih.gov/pubmed/15802482 10.1197/jamia.M1777 PMC1174889 15802482

[27] El-Kareh RE, Gandhi TK, Poon EG, Newmark LP, Ungar J, Orav EJ, et al. Actionable reminders did not improve performance over passive reminders for overdue tests in the primary care setting. Journal of the American Medical Informatics Association: JAMIA [Internet]. 2011 Apr 27 [cited 2011 Apr 27];18(2):160–3. Available from: http://www.ncbi.nlm.nih.gov/pubmed/21278102 10.1136/jamia.2010.003152 PMC3116255 21278102

[28] Hysong SJ. Meta-analysis: audit and feedback features impact effectiveness on care quality. Medical care [Internet]. 2009 Mar 1 [cited 2009 Mar 1];47(3):356–63. Available from: http://www.ncbi.nlm.nih.gov/pubmed/19194332 10.1097/MLR.0b013e3181893f6b PMC4170834 19194332

[29] Loo TS, Davis RB, Lipsitz LA, Irish J, Bates CK, Agarwal K, et al. Electronic medical record reminders and panel management to improve primary care of elderly patients. Archives of internal medicine [Internet]. 2011 Sep 26 [cited 2011 Sep 26];171(17):1552–8. Available from: http://www.ncbi.nlm.nih.gov/pubmed/21949163 10.1001/archinternmed.2011.394 21949163

[30] Bell CM, Levinson W. Pay for performance: learning about quality. CMAJ: Canadian Medical Association journal = journal de l’Association medicale canadienne [Internet]. 2007 Jun 5 [cited 2007 Jun 5];176(12):1717–9. Available from: http://www.ncbi.nlm.nih.gov/pubmed/17548385 10.1503/cmaj.070472 PMC1877846 17548385

[31] Petersen LA, Woodard LD, Urech T, Daw C, Sookanan S. Does pay-for-performance improve the quality of health care? Annals of internal medicine [Internet]. 2006 Aug 15 [cited 2006 Aug 15];145(4):265–72. Available from: http://www.ncbi.nlm.nih.gov/pubmed/16908917 10.7326/0003-4819-145-4-200608150-00006 16908917

[32] Scott A, Sivey P, Ait Ouakrim D, Willenberg L, Naccarella L, Furler J, et al. The effect of financial incentives on the quality of health care provided by primary care physicians. The Cochrane database of systematic reviews [Internet]. 2011 Sep 7 [cited 2011 Sep 7];(9):CD008451. Available from: http://www.ncbi.nlm.nih.gov/pubmed/21901722 10.1002/14651858.CD008451.pub2 21901722

[33] Chambers CV, Balaban DJ, Carlson BL, Grasberger DM. The effect of microcomputer-generated reminders on influenza vaccination rates in a university-based family practice center. The Journal of the American Board of Family Practice [Internet]. 1991 Feb 1 [cited 1991 Feb 1];4(1):19–26. Available from: http://www.ncbi.nlm.nih.gov/pubmed/1996510 1996510

